# A novel perspective of calvarial development: the cranial morphogenesis and differentiation regulated by dura mater

**DOI:** 10.3389/fcell.2024.1420891

**Published:** 2024-06-24

**Authors:** Danya Li, Xuxi Jiang, Jing Xiao, Chao Liu

**Affiliations:** Department of Oral Pathology, School of Stomatology, Dalian Medical University, Dalian, China

**Keywords:** cranium, suture mesenchymal cells, dura mater, cranial neural crest cells, osteogenesis

## Abstract

There are lasting concerns on calvarial development because cranium not only accommodates the growing brain, but also safeguards it from exogenous strikes. In the past decades, most studies attributed the dynamic expansion and remodeling of cranium to the proliferation of osteoprecursors in cranial primordium, and the proliferation of osteoprogenitors at the osteogenic front of cranial suture mesenchyme. Further investigations identified series genes expressed in suture mesenchymal cells as the markers of the progenitors, precursors and postnatal stem cells in cranium. However, similar to many other organs, it is suggested that the reciprocal interactions among different tissues also play essential roles in calvarial development. Actually, there are increasing evidence indicating that dura mater (DM) is indispensable for the calvarial morphogenesis and osteogenesis by secreting multiple growth factors, cytokines and extracellular matrix (ECM). Thus, in this review, we first briefly introduce the development of cranium, suture and DM, and then, comprehensively summarize the latest studies exploring the involvement of ECM in DM and cranium development. Eventually, we discussed the reciprocal interactions between calvarium and DM in calvarial development. Actually, our review provides a novel perspective for cranium development by integrating previous classical researches with a spotlight on the mutual interplay between the developing DM and cranium.

## 1 Introduction

As one of the most common congenital malformations in human newborns, calvarial malformations are manifested as craniosynostosis resulting from the premature closure of cranial sutures, found in Saethre-Chotzen Syndrome, etc. (Choi et al., 2021; Yu et al., 2021), and enlarged fontanelles caused by delayed suture closure, such as in Cleidocranial Dysplasia (Pan et al., 2017; Thaweesapphithak et al., 2022). Currently, the osteogenic differentiation at the ossifying front and stem cell properties of sutures attract most concerns on calvarial development (Chen et al., 2020; Yu et al., 2021). However, there are growing evidences implicating exquisite inter-cell or tissue orchestrations during calvarial development (Ibarra et al., 2021), yet the underlying mechanisms are unknown.

## 2 The development of cranium

### 2.1 The constitution and origins of cranium

**FIGURE 1:**
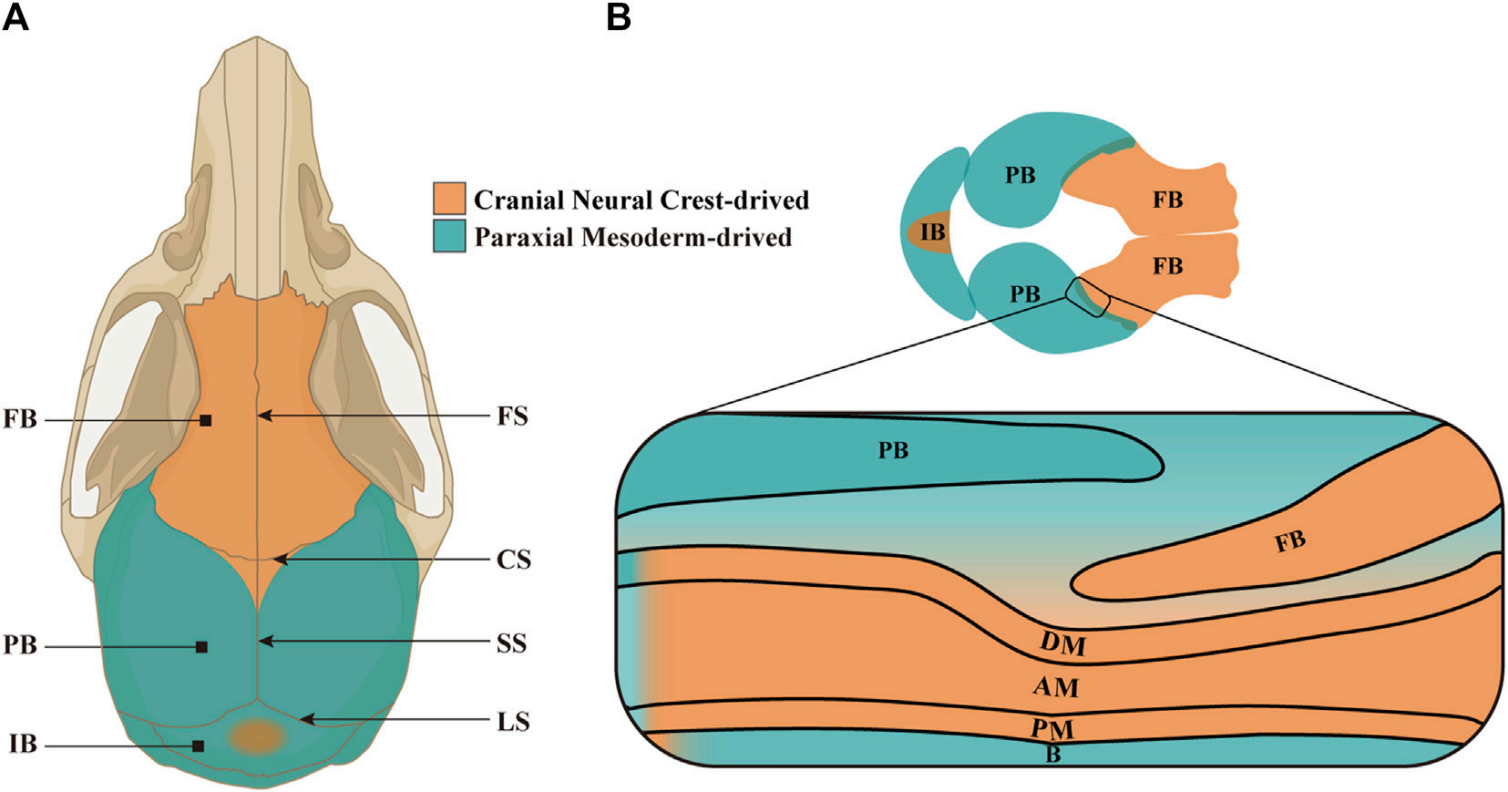
The constitution and origins of craniums and CS. **(A)** Schematic model depicting the structure and origins of adult mouse cranium in a vertical view. The paired FBs are derived from CNCCs (in orange), whereas the paired PBs and single IB from PMs (in green) and CNCCs (in orange) in a mosaic pattern. The four principal sutures in cranial vault are FS between paired FBs, CS formed by the PB covering the FB, SS between PBs, and LS between PBs and IB. **(B)** Schematic model portraying the composition and origins of embryonic mouse cranium in a vertical view, and an enlarged CS in lateral view. Notably, the meninges located below the anterior half of PB originate from CNCCs, while the meninges below posterior half from PMs. FB, frontal bone; PB, parietal bone; IB, interparietal bone; FS, frontal suture; CS, coronal suture; SS, sagittal suture; LS, lambdoid suture; DM, dura mater; AM, arachnoid mater; PM, pia mater; B, brain.

Confined by frontonasal, frontozygomatic and temporozygomatic sutures, cranium is made up of paired frontal bones (FBs) and parietal bones (PBs), as well as one interparietal bone (IB, also known as occipital bone in humans) (Jin et al., 2016; White et al., 2021). These flat bones are connected by cranial sutures which consist of the mesenchyme between two opposing edges of cranial bones (Li et al., 2021). There are four principal sutures on cranial vault, namely, frontal suture (FS), sagittal suture (SS), coronal suture (CS) and lambdoid suture (LS). In the first month after birth, prior to the closure of mouse posterior frontal suture (PFS), a rhombic gap termed anterior fontanelle is formed at the intersection of CS and SS (Sahar et al., 2005; Stanton et al., 2022). Putatively, it is regarded that FBs, the central portion of IB, SS and FS originate from cranial neural crest cells (CNCCs), whereas PBs, peripheral portion of IB and CS from paraxial mesodermal cells (PMs). Actually, by multiple genetic reporter mice, it is demonstrated that the cranium, especially PBs, CS and SS, are built by CNCCs and PMs in a mosaic pattern (Jiang et al., 2002; McBratney-Owen et al., 2008; Henrique et al., 2012; Doro et al., 2019; White et al., 2021; Doro et al., 2023). Such mosaic CNCC-PM pattern in CS is maintained by *En1* and *Twist1* expression, and leads to craniosynostosis due to premature closure of lateral CS when the mosaic pattern is disrupted (Ting et al., 2009; Deckelbaum et al., 2012) (Figure 1).

### 2.2 The cranium morphogenesis

**FIGURE 2:**
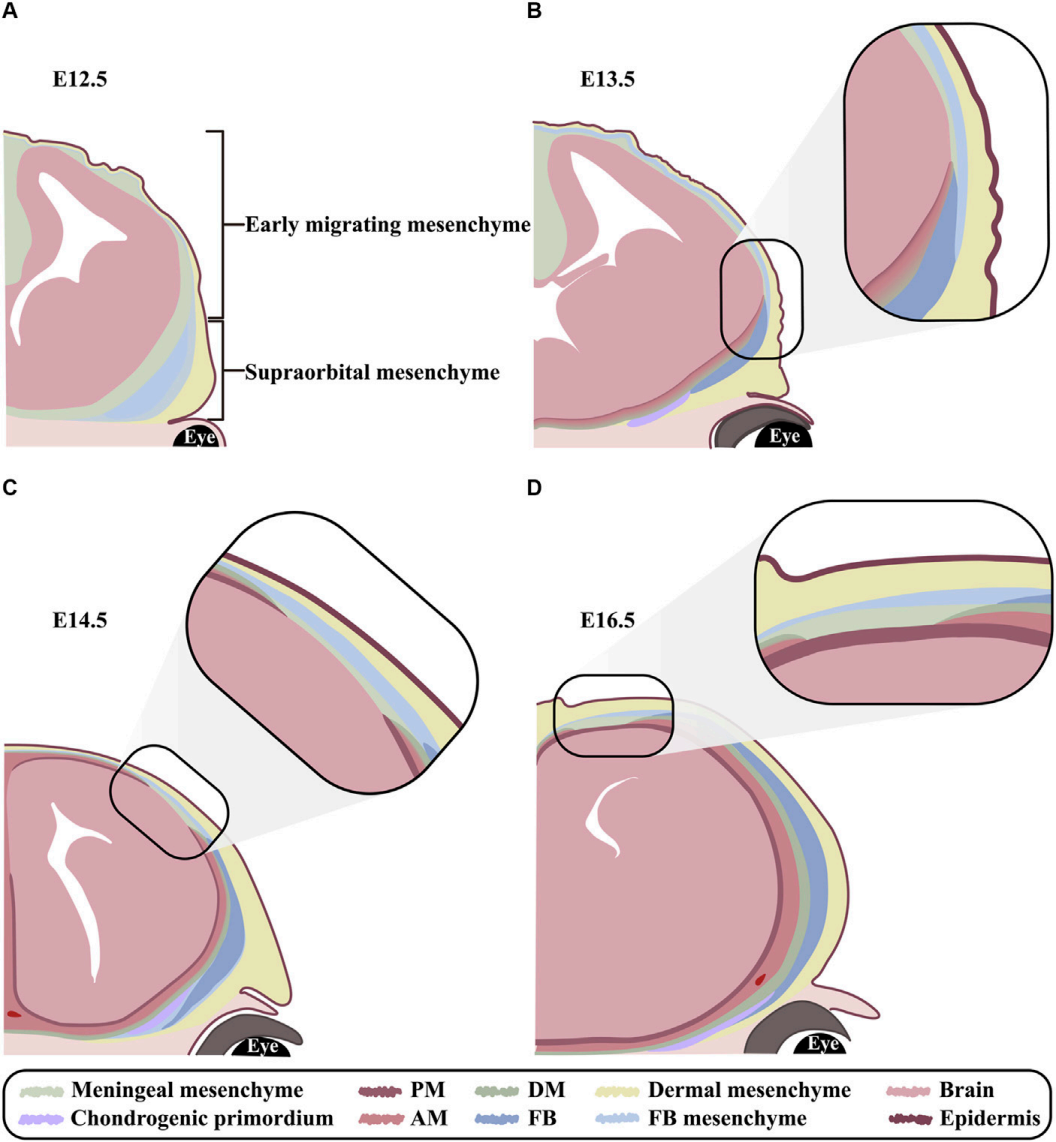
The orchestrated development of FB and meninges **(A–D)** Schematic models exhibiting FB and meningeal development at E12.5, E13.5, E14.5 and E16.5 in coronal view. **(A)** At E12.5, the cranial mesenchyme overlying brain is divided into EMM and SOM. EMM are specified into outer dermal mesenchyme and inner meningeal mesenchyme. In contrast, SOM are specified into the outmost dermal, the middle frontal, and the innermost meningeal mesenchyme. Notably, the condensed mesenchyme in frontal region is known as frontal primordium. **(B)** At E13.5, although the meningeal mesenchyme in SOM is committed into DM, arachnoid mater and pia mater from basis to apex, yet the three layers of meninges remain indiscernible until E14.5. Meanwhile, the osteogenesis commences in frontal primordium and expands apically. **(C)** At E14.5, the meningeal mesenchyme in EMM is committed to DM, arachnoid mater and pia mater from the outer to inner along apical-basolateral direction. Meanwhile, frontal lamella grows apically in accordance with meningeal differentiation. **(D)** At E16.5, both frontal and meningeal mesenchyme are differentiating in a lateral to apical orientation. PM, pia mater; AM, arachnoid mater; DM, dura mater; FB, frontal bone; FB mesenchyme, frontal bone mesenchyme.

Between embryonic day 8.5 (E8.5) and E9.5, CNCCs and PMs begin to migrate from mid-hindbrain towards the anterior and posterior of supraorbital arch, respectively, and form a cranial mesenchymal sheath enveloping brain at E10.5. The cranial mesenchymal sheath is known as primary meninx, and divided into the basolateral supraorbital mesenchyme (SOM) and the apical early migrating mesenchyme (EMM). As a vital morphogenic and ossification center for both FBs and PBs, SOM eventually generates dermis, cranium and meninges. While EMM is fated only to dermal and meningeal layers. At E12.5, the rudiments of PBs and FBs between the dermal and meningeal layers in SOM initiate mesenchymal condensation and osteogenic commitment (Jiang et al., 2002; Yoshida et al., 2008; Roybal et al., 2010; Dasgupta et al., 2019; Salhotra et al., 2020; Vu et al., 2021) (Figure 2A). Since the osteogenesis of PBs and FBs is accomplished through intramembranous ossification, a cascade of transcription factors, including *Msx1* and *Msx2*, *Runx2* and *Osx*, are activated directly in the condensed mesenchyme and osteogenic precursors (Han et al., 2007; Deckelbaum et al., 2012). From E13.5 on, the proliferating osteogenic precursors in the leading edges of PB and FB primordia migrate upwardly along the gradient of Fibronectin (Fn1) in dermal and meningeal mesenchyme (Yoshida et al., 2008; Ting et al., 2009; Roybal et al., 2010; Feng et al., 2023). In addition, BMPs emanating from meningeal mesenchyme also persistently promote cell proliferation and differentiation in the basolateral calvarial rudiments to fulfill the vertical expansion of FB and PB lamellae (Machida et al., 2014) (Figure 2B). The growth of calvarial lamella is dominantly determined by the quantities of osteoprogenitors in primordium and pre-osteoblasts at osteogenic fronts (OFs) (Lana- Elola et al., 2007). At E14.0-E14.5, the primary ossification center is emerging in the sheet-like lamella stretching vertically, which reaches the apex at E18.5 (Iseki et al., 1997; Han et al., 2007; Yoshida et al., 2008) (Figures 2C, D). Furthermore, ECM plays critical roles in the osteogenic differentiation of calvarial progenitors by regulating the activities of multiple signaling pathways, because proteoglycans in cranial mesenchyme show differential affinity to growth factors according to the different glycosaminoglycan (GAG) chains (Bhat et al., 2011). Our previous studies showed that knocking out *Family with sequence similarity member 20-b* (*Fam20b*) in mouse CNCCs also resulted in persistent potency of sutures and craniosynostosis by impairing GAG chain synthesis (Liu et al., 2018), implicating the role of GAG chains in mediating signaling activity in CNCC derived cranium.

### 2.3 Cranial suture mesenchyme

Cranial sutures not only cushion mechanical impacts to protect the soft cerebrum, but also facilitate the osteogenesis of calvarial bones to accommodate the growing brain (Zhao et al., 2015; Zhao et al., 2023). Previous researches have demonstrated that the differentiation of cranial suture mesenchymal cells (CSMCs) in SS into osteoblasts contributed to PB morphogenesis (Lana-Elola et al., 2007; Angelozzi et al., 2022). CSMCs are embedded by the overlying suprasutural mesenchymal layer and the underlying periosteal dura layer (Figure 1B). According to the cell atlas of CS at E15.5, CSMCs are uncommitted progenitors, whereas the pre-osteoblasts in OFs are proximal to CS, and the osteoblasts and osteocytes in OFs distal from CS (Farmer et al., 2021). During cranium morphogenesis, CSMCs along the OFs undergo osteogenic differentiation, but remain undifferentiated at the core of sutures to guarantee the lifetime patency of cranial sutures (Farmer et al., 2021; Li et al., 2021).

There are four well-recognized markers, *Gli1*, *Prrx1*, *Axin2* and *Ctsk*, identifying CSMCs with distinct characteristics. *Gli1* ^*+*^ CSMCs in cranial sutures are detected adjacent to OFs as early as E15.5, and extensively scattered in periosteum, DM and suture mesenchyme from P0 (except for PFS at P8 and P30). Then, *Gli1* ^*+*^ CSMCs are gradually limited to the mid-suture region in 1 month postnatally. *Gli1* ^*+*^ CSMCs prefer to differentiate into osteochondral progenitors which generate OFs, periosteum and DM. Adult mice with partial ablation of *Gli1* ^*+*^ cells have prematurely fused CS and anterior FS, as well as retarded repair for calvarial injury, indicating the property of osteogenic stem cells in *Gli1* ^*+*^ CSMCs (Zhao et al., 2015; Holmes et al., 2020; Farmer et al., 2021). *Prrx1* ^*+*^ CSMCs are intensively detected in CS and OFs at E15.5, but only in PFS, CS, SS, and LS, not in other sutures, DM or periosteum after birth. Despite the decline with age, *Prrx1* ^*+*^ CSMCs contribute to CSMC niche, periosteum and neighboring OFs. Interestingly, global ablation of embryogenic *Prrx1* ^*+*^ cells causes various degrees of FB and PB dysplasia, yet global ablation of postnatal cells does not. Thus, *Prrx1* ^*+*^ CSMCs are suggested to represent a quiescent population which is crucial to maintain suture patency, and capable of commence osteoblast differentiation under sufficient stimulation (Wilk et al., 2017; Farmer et al., 2021). Notably, *Axin2* ^*+*^ CSMCs located in E18.5 CS are dispersed in patent PFS, CS, SS and LS from P5, and progressively confined to the center of sutures by P9. Normally, *Axin2* ^*+*^ CSMCs in PFS are reduce gradually in coordination with PFS closure by P28. Besides in patent cranial sutures, *Axin2* ^*+*^ CSMCs and their offsprings are found surrounding OFs, periosteum and DM. Unexpectedly, *Axin2* homozygotic knockout mice exhibit premature fusion in PFS, demonstrating the role of *Axin2* ^*+*^ CSMCs in maintaining suture stem cell niche (Maruyama et al., 2010; Alman et al., 2013; Maruyama et al., 2016; Holmes et al., 2020). Since it is well established that *Ctsk* ^*+*^ periosteal stem cells are responsible for intramembranous ossification in long bones, the role of *Ctsk* ^*+*^ CSMCs in cranium development also attracts concerns. *Ctsk* ^*+*^ CSMCs are found in sutures at E16.5, and dispersed in suture mesenchyme, periosteum and bone marrow of OFs at P7. Postnatal inactivation of *Ctsk* ^*+*^ CSMCs resulted in premature fusion of cranial sutures and hypomineralization by converting CSMCs into ectopic chondrocytes (Debnath et al., 2018; Bok et al., 2023). Thus, *Ctsk* most likely maintains the osteogenic specification in postnatal CSMCs by suppressing the chondrogenic fate.

## 3 The development of dura mater

### 3.1 Anatomy and origins of dura mater

Meninges are multilayered structures encasing the central nervous system, composed of fibroblasts, blood cells, lymphatic capillaries and immune cells. Meninges are classified into DM, arachnoid mater (AM) and pia mater from the outer to inner side (Nelson, 2009; Decimo et al., 2020). As a dense collagenous membrane beneath cranium, DM is further divided into endosteal dura attached to calvarial bone, and meningeal dura to AM, between which are dural venous sinus (Patel and Kirmi, 2009; Adeeb et al., 2012; DeSisto et al., 2020). As a spongy connective tissue located between DM and pia mater, AM is comprised of a numerous collagens and fibroblasts (Decimo et al., 2012). In contrast, pia mater is a highly vascularized and fragile sheath tightly adhering to brain (Adeeb et al., 2013). All three meningeal layers covering forebrain originate from CNCCs, differing from those covering midbrain and hindbrain, which derive from PMs. Correspondingly, the meninges beneath FBs and IB are exclusively from CNCCs and PMs, respectively, whereas the meninges covered by PBs are originated both from PMs (the middle and posterior portion) and CNCCs (the anterior portion along CS) (Yoshida et al., 2008; Dasgupta and Jeong, 2019) (Figure 1B).

### 3.2 Morphogenesis of dura mater

At E10.5, both the EMM and SOM of primary meninx are subdivided into a condensed outer layer, and a reticular inner layer containing meningeal progenitors. Upon E13.5, the meningeal progenitors in SOM are specified into the outmost DM, the middle AM and the innermost pia mater from basis to apex (Figure 2B). In contrast, the meningeal progenitors in EMM initiate meningeal commitment along apical-basolateral orientation at E14.5 (Bjornsson et al., 2015; Dasgupta et al., 2019; DiNuoscio and Atit, 2019; Vu et al., 2021) (Figure 2C). The layers of DM, AM and pia mater are indiscernible until E14.5, and completely distinguishable at E19.5 (Bifari et al., 2015; Dasgupta and Jeong, 2019) (Figures 2C, D). Currently, *Foxc1*, which is robustly expressed in the entire meningeal and osteogenic mesenchyme from E11.0, and eventually confined in meninges and FB, is regarded as the determinator in meningeal specification. *Foxc1* null mice exhibited unspecified meningeal mesenchyme apically, as well as the compressed AM and DM basolaterally, which was attributed to the disrupted cytoskeleton (Rice et al., 2003; Sun et al., 2013; Machida et al., 2014). Moreover, as a non-osteogenic mesenchyme, the apical meningeal precursors of EMM exhibited chondrogenic potential with the enhanced *Dlx5* expression in CNCCs (Vu et al., 2021). Similarly, inactivation of Wnt/β-catenin signaling in cranial mesenchyme converts calvarial mesenchyme into ectopic cartilage, and impairs the apical expansion of DM from basolateral SOM (DiNuoscio and Atit, 2019), indicating that both basolateral SOM and apical EMM meningeal populations require Wnt/β-catenin signaling during morphogenesis.

### 3.3 The regulation of dura mater on cranial development

**FIGURE 3:**
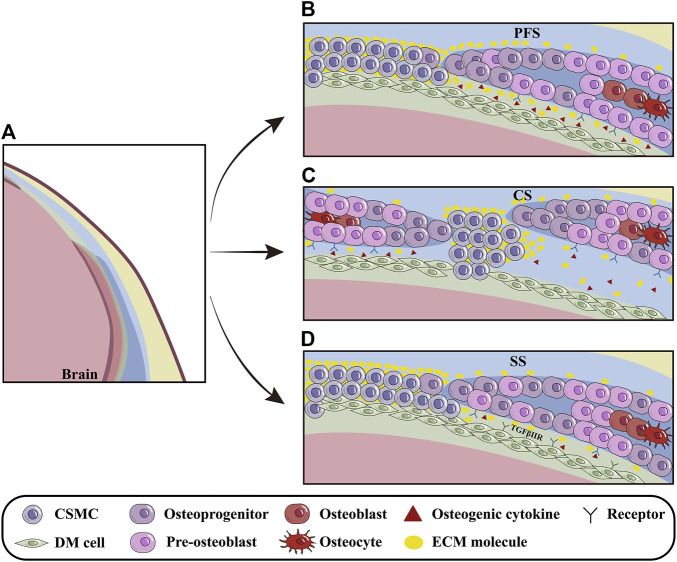
The influence of DM on cranial osteoprecursors at OFs. **(A)** Schematic models depicting the regulation of DM on the cranial osteoprecursors at OFs during apical growth. **(B)** The PFS, the sole cranial suture capable of closure, is rich in osteogenic cytokines and ECM molecules such as BMP2/4/7, SULF1 and others. The basolateral-apical ECM gradient (especially Fn1) provides migratory guidance for calvarial osteoprecursors at OFs. **(C)** In CS, osteogenic cytokines produced by DM, such as TGFβ2 and FGF2, regulate pre-osteoblasts and promote closure. **(D)** Compared to CNCC-derived PFS, PM-derived SS contains fewer osteogenic factors and ECM molecules, most likely due to different origins of calvarial bones and meninges during suturogenesis. Furthermore, TGF signaling can indirectly modulate PB osteogenesis during meningeal development. PFS, posterior frontal suture; CS, coronal suture; SS, sagittal suture.

Meningeal development is dynamically orchestrated with cranium morphogenesis through the interplays among OFs, sutural mesenchyme and the underlying meninges (Vivatbutsiri et al., 2008) (Figure 3A). Opperman *et al.* revealed that the soluble factors emanating from DM maintained CS patency, which allowed FB and PB expansion to accommodate enlarging brain (Opperman et al., 1993). It is implicated that the DM beneath PFS even secreted much more osteogenic growth factors and ECM molecules than that beneath SS, which makes the earlier closure of PFS to specifically determine the ratio of viscerocranium to neurocranium (Greenwald et al., 2000; White et al., 2021). The discrepancy in ECM compositions between the CNCC-derived and PM-derived DMs is most likely attributed to their different origins, because the CNCC-derived FS expresses the higher concentration of ECM involved in CNCC migration and cell-cell communication, such as SULF1 and ICAM1, compared to PM-derived SS (Homayounfar et al., 2015) (Figures 3B, D).

During embryonic stage, DM is essential for cranial osteogenesis because deletion of *Tgfbr2* in CNCCs leads to hypoplasia of CNCC-derived DM, which in turn, impairs the osteogenesis of PM-derived PBs (Ito et al., 2003). Furthermore, the compressed DM and AM in conventional *Foxc1* knock-out mice suppressed pre-osteoblast proliferation by up-regulating *Msx2* and down-regulating *Bmp2/4/7* in OFs, implicating that the hypoplastic meninges impact the apical expansion of cranium *via* BMP signaling (Sun et al., 2013; Machida et al., 2014). With the aid of single-cell transcriptome analysis, *Tgfβ2*, *Fgf2*, *Gdf10* and *Ctgf64* expressed in the endosteal dura are suggested to be involved in CS closure by regulating pre-osteoblasts (Farmer et al., 2021). Additionally, the *Hhip* ^*+*^ cells within CS are also predicted by CellPhoneDB to interact closely with DM and osteogenic cells (Holmes et al., 2021) (Figures 3B–D).

In the developing cranium, Fn1 distribution in cranial mesenchyme forms a basolateral-apical gradient. With the basolateral to apical growth of calvarial primordium, the Fn1 distribution is diminished in the extending OFs and retreats apically. Abrogation of *Fn1* in cranial mesenchyme leads to insufficient apical extension of FBs and craniosynostosis in CS, suggesting that Fn1 provides migratory guidance for the apical expansion of calvarial osteoprecursors (Feng et al., 2023) (Figure 3C). It remains unknown whether Fn1 produced by DM acts as a substrate for the migratory osteogenic progenitors of cranium.

Postnatally, meninges are indispensable for cranium development by releasing osteogenic growth factors, cytokines and ECM (Decimo et al., 2020). Additionally, DM is capable of not only secreting BMP2 to induce the differentiation of exogenous human adipose-derived stromal cells into osteoblasts to repair calvarial damage by activating canonical BMP signaling, but also emanating BMP4, together with the BMP2 from pre-osteoblasts, to re-model the cerebral venous diameter and branches (Levi et al., 2011; Tischfield et al., 2017). Meanwhile, cranial osteoprogenitors also regulate the formation of lymphatics in meninges by secreting vascular endothelial growth factor-C (Ma et al., 2023).

## 4 Discussion

Up to date, it is putatively regarded that calvarial development depends on the interplays with surrounding tissues, especially DM, which emanates numerous osteogenic factors and ECM to influence OFs and CSMCs (Levi et al., 2011; Homayounfar et al., 2015; Tischfield et al., 2017; Decimo et al., 2020; Holmes et al., 2021; Feng et al., 2023). However, the molecular mechanism underlying these spatiotemporal interactions, as well as the dynamic cellular behaviors, remain poorly understood. For example, it is still in debates that whether the basolateral and apical meningeal populations play distinct roles in the osteogenesis of cranial primordium. Moreover, since ECM is crucial for the repairing of cranial defects and the homeostasis of CSMC niche, the exact roles of proteoglycans and GAG chains in meningeal and calvarial development, which mediates the interactions between DM and cranium, still require further investigations (Mansour et al., 2017; Yamaguchi et al., 2021). However, the accumulated transcriptomic and proteomic knowledge at single cell resolution would deepen our understanding on the molecular mechanisms involved in the meningeal and cranial development and interplay, through which not only the perspective on cranial development and CSMC homeostasis, but also the regeneration strategy for cranial trauma and congenital calvarial malformations, would be shed light on.
